# Reporting of Patient Experience Data on Health Systems’ Websites and Commercial Physician-Rating Websites: Mixed-Methods Analysis

**DOI:** 10.2196/12007

**Published:** 2019-03-27

**Authors:** Tara Lagu, Caroline M Norton, Lindsey M Russo, Aruna Priya, Sarah L Goff, Peter K Lindenauer

**Affiliations:** 1 Baystate Health Institute for Healthcare Delivery and Population Science and Department of Medicine University of Massachusetts Medical School-Baystate Springfield, MA United States; 2 College of Natural Sciences University of Massachusetts Amherst, MA United States; 3 School of Public Health and Health Sciences University of Massachusetts Amherst, MA United States

**Keywords:** physician reviews, social networking, public reporting

## Abstract

**Background:**

Some hospitals’ and health systems’ websites report physician-level ratings and comments drawn from the Consumer Assessment of Healthcare Providers and Systems surveys.

**Objective:**

The aim was to examine the prevalence and content of health system websites reporting these data and compare narratives from these sites to narratives from commercial physician-rating sites.

**Methods:**

We identified health system websites active between June 1 and 30, 2016, that posted clinician reviews. For 140 randomly selected clinicians, we extracted the number of star ratings and narrative comments. We conducted a qualitative analysis of a random sample of these physicians’ narrative reviews and compared these to a random sample of reviews from commercial physician-rating websites. We described composite quantitative scores for sampled physicians and compared the frequency of themes between reviews drawn from health systems’ and commercial physician-rating websites.

**Results:**

We identified 42 health systems that published composite star ratings (42/42, 100%) or narratives (33/42, 79%). Most (27/42, 64%) stated that they excluded narratives deemed offensive. Of 140 clinicians, the majority had composite scores listed (star ratings: 122/140, 87.1%; narrative reviews: 114/140, 81.4%), with medians of 110 star ratings (IQR 42-175) and 25.5 (IQR 13-48) narratives. The rating median was 4.8 (IQR 4.7-4.9) out of five stars, and no clinician had a score less than 4.2. Compared to commercial physician-rating websites, we found significantly fewer negative comments on health system websites (35.5%, 76/214 vs 12.8%, 72/561, respectively; *P*<.001).

**Conclusions:**

The lack of variation in star ratings on health system sites may make it difficult to differentiate between clinicians. Most health systems report that they remove offensive comments, and we notably found fewer negative comments on health system websites compared to commercial physician-rating sites.

## Introduction

Approximately 60% of US consumers report that online reviews are either somewhat or very important when choosing a physician [1]. Of patients who have used reviews to choose a physician, 52% report that they have chosen not to see a given physician because of review content [2]. However, commercial physician-rating websites (designed similarly to websites that review restaurants and hotels) are difficult to use and have few reviews per physician [3,4]. There is an ongoing effort by leaders in the field to systematically collect and publicly report patient narratives about individual physicians, but the potential for widespread implementation of these initiatives remains unclear [5-10].

In response to this gap and as a mechanism to increase market share, some hospitals and health systems across the United States have begun to compile and report physician-level ratings and comments drawn from the Consumer Assessment of Healthcare Providers and Systems (CAHPS) surveys [11]. Typically, a health system engaging in such an effort summarizes CAHPS data through a composite score (hereafter called a “star rating”) and posts this score on the physician’s biographical webpage within the health system website. Many health systems also post patients’ narrative responses to open-ended questions (eg, “What did this clinician do well?” and “What could this clinician do better?”). Although these initiatives have received attention in both the medical and lay press [11,12], there has been only one description of the phenomenon in the medical literature [13]. The number of US health systems that are participating in these efforts is unknown, the content of reviews on health systems’ sites has not been described (nor has it been compared to the previously existing narrative content about physicians reviews on commercial rating websites), and the implications for patient experience and quality improvement activities have yet to be explored. Therefore, we aimed to characterize the content of health systems’ webpages that report these results, including numbers of star ratings and narrative reviews per clinician, and to compare the content of narrative comments drawn from commercial physician-rating websites.

## Methods

### Data Sources, Website Identification, Search Characteristics, and Hospital Characteristics

The organizations that first published compiled patient experience data were large systems associated with a hospital or hospitals [12]. Therefore, we obtained data from the Center for Medicare and Medicaid Services’ Hospital Compare website and examined the websites of all listed hospitals [14]. For each entity, we verified the name and street address and then determined if reviews were present. To identify participating health systems that were not associated with a hospital, we obtained a published online list of health systems [14] known to post reviews. We examined all sites on this list and supplemented our search using a previously described method for systematically searching Google (eg, “doctor reviews”) [4].

We included websites that were functional (ie, had working links) between June 1 and 30, 2016, and had at least one clinician with star ratings or narrative comments. We used American Hospital Association data to generate hospital descriptive statistics (characteristics of health systems without a participating hospital were not captured). Because all included data were publicly available, the Baystate Institutional Review Board deemed that this study did not constitute human subjects research.

### Website Structure and Extraction of Physician-Review Data

Using an extraction method described previously, we created an a priori list of website classification criteria to describe included websites [4]. In brief, this included elements such as methods that could be used to search for clinicians (eg, specialty, name, location), description of methods used to remove “offensive” reviews, and review structure (eg, star ratings vs narratives). Three authors (TL, CN, LR) then reviewed the content of the websites and added classification categories as appropriate. Two authors then completed a final review of included websites (CN, LR).

To examine a sample of clinician reviews, we obtained lists of clinicians for 14 of the identified health systems from the National Research Corporation (NRC) [15]. NRC is a for-profit consulting firm focused on improving patient experience and health system brand loyalty. We limited our sample to health systems for which we could obtain lists of clinicians because without a list we were unable to randomly select clinicians. We used random number generation to select 10 clinicians from each of the 14 identified health systems. For each clinician, we then extracted the number of star ratings, number of narrative comments, and total or average star rating. We quantified occurrences of each type of review using descriptive statistics (frequencies and percentages).

### Quantitative and Qualitative Analysis of Narrative Reviews

We used qualitative methods to examine narrative reviews for included physicians. We selected the five most recent narratives from each of the 140 (10 clinicians from 14 health systems) randomly selected clinicians’ profiles (if there were fewer than five narratives, we took as many as were present). Beginning with themes described in related studies [4,16,17], we created a codebook. We then developed additional codes to capture themes and content that were not in the codebook. We repeated this iteratively until the team felt that the coding categories captured the major substantive content reviewed. After establishing 80% agreement, the researchers each completed coding independently (all reviews were double-coded) and met once more to reach an agreement on all codes, resolving differences by consensus. Applying directed qualitative content analysis methods [18,19], we then organized codes into pertinent major and minor themes. One author (CN) then checked for accuracy of coding and performed second-level coding to synthesize themes and content, which was reviewed with the other authors. We used descriptive statistics to describe the frequency with which major and minor themes occurred.

### Comparison of Narrative Reviews From Health System Websites to Those From Commercial Physician-Rating Websites

In a prior study of 600 physicians selected from three geographically diverse US cities, we collected more than 1800 narrative reviews from 28 different commercial physician-rating websites [3]. We conducted a simultaneous qualitative analysis of a randomly sampled set of 214 comments taken from these 28 commercial physician-rating sites and compared the results to those obtained from our qualitative analysis of comments from health systems’ websites. Using the codebook created for the analysis of reviews from health systems, the two coders coded commercial website reviews independently and then met to discuss discrepancies in coding. Investigators resolved differences in coding and updated the codebook using an iterative process. We continued this process until no new codes were identified in 10 sequential reviews, resulting in a comprehensive codebook that covered both commercial rating websites and health systems (Multimedia Appendix 1: codebook) and a comprehensive list of themes (Multimedia Appendix 2: themes). Two investigators (CN, LR) then independently coded the remaining reviews. We compared the percentage of reviews for each theme between health system websites and commercial rating websites using the chi-square test and the Fisher exact test. All analyses were performed using SAS version 9.4 (SAS Institute Inc, Cary, NC, USA).

## Results

### Website Identification, Search Characteristics, and Hospital Characteristics

From 4800 hospitals on Hospital Compare, we identified 161 hospitals (3.4%) that posted star ratings or narrative comments about clinicians. Many of these hospitals were affiliated hospitals within a larger system, so we collapsed the 161 hospitals into 36 health systems. Our search methods identified an additional eight health systems that were not associated with hospitals, which gave us a total of 42 health systems from 26 states. This represented approximately 7% of the 626 health systems in the United States [20]. All identified health systems published star ratings (42/42, 100%) and most published narrative reviews (33/42, 79%).

### Website Structure

No sites described their method for calculating star ratings. The majority (27/42, 64%) stated on their main page that they excluded narratives deemed inappropriate or offensive, but none explained how this process was conducted. Most allowed users to search for physicians by name (39/42, 93%), specialty (41/42, 98%), and location (31/42, 74%). Nearly half of included hospitals (79/169, 46.8%) had fewer than 200 beds (Table 1). More than a third (61/169, 36.1%) were located in the western region of the United States. Acute care hospitals made up the majority (145/169, 85.8%) of the sample.

### Quantity of Reviews

Of the randomly sampled 140 clinicians from 14 health systems, there were 21,332 quantitative reviews and 4723 narrative reviews. A majority of clinicians had reviews (star ratings: 122/140, 87.1%; narrative reviews: 114/140, 81.4%), with medians of 110 star ratings (interquartile range [IQR] 42-175) and 25.5 narratives (IQR 13-48) per clinician. Only one clinician in the sample did not have any reviews. In general, star ratings were quite high with little variation between physicians: the median rating was 4.8 (IQR 4.7-4.9) out of five stars. Of 140 physicians, none had a score of less than 4.2.

### Quantitative and Qualitative Analysis of Narrative Reviews

Using the five (or less if five were not available) most recent reviews from 140 clinicians from 14 health systems, we identified 561 health system narrative reviews for qualitative analysis. As described in Methods, we also analyzed 214 narrative comments previously randomly sampled from 600 physicians across 28 commercial physician-rating websites.[3] Themes that emerged from coding these two sets of data included general positive and negative comments about clinicians, clinician communication and interpersonal skills, technical skills, facility and office experience, patient care experience (independent of these other themes), descriptions of “reasons for seeking care,” and “extreme comments” (ie, long descriptions of very positive or negative experiences that did not fit well into other categories). Example quotes from these themes are given in Multimedia Appendix 3 (example quotations for identified themes).

### Comparison of Narrative Reviews From Health System Websites to Those From Commercial Physician-Rating Websites

Overall, the vast majority of comments were positive (642/775, 82.8% for all narratives), including 71.0% (152/214) of commercial rating websites comments and 87.3% (490/561) of health systems’ websites comments (*P*<.001). Negative comments were less common, but commercial rating sites had a greater proportion of negative reviews compared to health systems’ sites (35.5%, 76/214 vs 12.8%, 72/561, respectively; *P*<.001) (Table 2). Within subcategories of positive comments, there were some significant differences between the two types of websites. Commercial rating websites had significantly more “clinician communication and personal skills” positive comments (commercial rating sites: 127/214, 59.3%; health systems’ sites: 238/561, 42.4%; *P*<.001), more positive “clinician technical skills” comments (commercial rating sites: 74/214, 34.6%; health systems’ sites: 84/561, 15.0%; *P*<.001), and more “extremely positive” comments (commercial rating sites: 9/214, 4.2%; health systems’ sites: 4/561, 0.7%; *P*=.002) compared to health system websites, whereas health system websites had significantly more positive “patient care experience” comments than commercial websites (commercial rating sites: 12/214, 5.6%; health systems’ sites: 106/561, 18.9%; *P*<.001).

**Table 1:** Characteristics of 169 hospitals posting physician reviews.

Characteristic		Hospitals, n (%)
**Number of** **beds^a^**		
	<200	79 (46.8)
	200 to 400	43 (25.4)
	>400	25 (14.8)
Teaching^a^		20 (11.8)
**Region^a^**		
	Midwest	25 (14.8)
	Northeast	27 (16.0)
	South	34 (20.1)
	West	61 (36.1)
**Hospital type^b^**		
	Acute care	145 (85.8)
	Children’s	2 (1.2)
	Critical access	15 (8.9)

^a^22 of 169 hospitals did not have an American Hospital Association identifier.

^b^7 of 169 hospitals did not have their type/ownership recorded.

**Table 2:** Comparison of occurrence of themes between health systems’ sites and commercial rating sites.

Themes		Total, n (%)	Commercial rating sites, n (%)	Health systems’ sites, n (%)	*P* value^a^
Sites		775 (100)	214 (27.6)	561 (72.4)	
**Positive themes**					
	Overall positive comments	642 (82.8)	152 (71.0)	490 (87.3)	<.001
	General positive comments about clinicians (great doctor, very good, would recommend, satisfied with care from provider)	345 (44.5)	89 (41.6)	256 (45.6)	.31
	Clinician communication and interpersonal skills	365 (47.1)	127 (59.3)	238 (42.4)	<.001
	Clinician technical skills	158 (20.4)	74 (34.6)	84 (15.0)	<.001
	Facility or office experience and staff characteristics	127 (16.4)	31 (14.5)	96 (17.1)	.38
	Patient care experience	118 (15.2)	12 (5.6)	106 (18.9)	<.001
	Reason for seeking care	5 (0.6)	2 (0.9)	3 (0.5)	.62^b^
	Extremely positive	13 (1.7)	9 (4.2)	4 (0.7)	.002^b^
**Negative themes**					
	Overall negative comments	148 (19.1)	76 (35.5)	72 (12.8)	<.001
	General negative comments about clinicians (would not recommend)	12 (1.5)	11 (5.1)	1 (0.2)	<.001^b^
	Clinician communication and interpersonal skills	72 (9.3)	40 (18.7)	32 (5.7)	<.001
	Technical skills	35 (4.5)	27 (12.6)	8 (1.4)	<.001
	Facility or office experience and staff characteristics	66 (8.5)	38 (17.8)	28 (5.0)	<.001
	Patient care experience	24 (3.1)	16 (7.5)	8 (1.4)	<.001
	Feedback about survey	7 (0.9)	0 (0)	7 (1.2)	—
	Extremely negative	13 (1.7)	13 (6.1)	0 (0)	—
**Neutral themes**					
	Neutral patient experience	86 (11.1)	22 (10.3)	64 (11.4)	.65

^a^All chi-square tests, except where noted.

^b^Fisher exact test.

In contrast, commercial websites had a higher percentage of negative comments across nearly all themes. For example, commercial rating websites had more negative “clinician communication and interpersonal skills” comments (commercial rating sites: 40/214, 18.7%; health systems’ sites: 32/561, 5.7%; *P*<.001), “technical skills” comments (commercial rating sites: 27/214, 12.6%; health systems’ sites: 8/561, 1.4%; *P*<.001), more “facility/office experience and staff characteristics” comments (commercial rating sites: 38/214, 17.8%; health systems’ sites: 28/561, 5.0%; *P*<.001), “patient care experience” comments (commercial rating sites: 16/214, 7.5%; health systems’ sites: 8/561, 1.4%; *P*<0.001), and more “extremely negative” comments (commercial rating sites: 13/214, 6.1%; health systems’ sites: 0/561, 0.0%) compared to health systems’ websites.

## Discussion

The phenomenon of health systems publishing systematically collected patient experience data about individual clinicians has been hailed as a triumph for transparency and patient-centeredness, but the scope and content of the narratives and reviews on health systems’ sites have not been previously described [11,12]. After a comprehensive search, we identified 42 of 626 health systems nationwide (7%) that were early adopters of this practice. Most clinicians’ pages had many reviews (both star reviews and narratives), which gives them an important advantage over existing commercial physician-rating websites [3]. However, most clinicians also had near-perfect star ratings, with 75% of physicians having a score of between 4.7 and 4.9 stars out of 5, and a minimum star rating of 4.2. Furthermore, we observed, similar to published literature [4,21], that narratives from both commercial rating websites and health systems’ websites were mostly positive (with similar percentages of positive responses across both types of sites), and themes that emerged were similar to themes seen in other studies that have examined review content [4,22,23]. Across a range of subthemes, we observed a statistically significantly smaller number of negative reviews for individual clinicians on health system sites when compared to commercial physician-rating sites.

There are several important implications to our findings. First, the narrow range of star ratings (the majority were in the 4.7 to 4.9 out of maximum 5) may limit the ability of patients to differentiate between clinicians using only star ratings on health systems’ websites. The tightly clustered distribution of scores near 5.0 may be the result of the fact that health systems calculate the composite score from the CAHPS multiquestion survey, and the majority of responses to CAHPS questions are either “usually” or “always” [5]. Less than 5% of respondents choose “never” for any CAHPS category [5]. It has also been previously reported in the literature that most online ratings for physicians are quite high [24]. Notably, we did not find any health systems that explained their methods for calculating the composite metric of “number of stars.”

Second, the large number of reviews and narratives on health systems’ websites (medians of 110 star ratings and 25 narratives for each physician) may address the gap between patients’ desire for reviews [1,2] and the previously reported lack of narrative and star reviews on physician-rating websites.[3,4]. Further, the large numbers of reviews we found may address some of the concerns about lack of an adequate sample (because health systems can also wait to post reviews until a sufficient number are collected or can use older surveys to increase the numbers of reviews and narratives) [12,25]. However, it has been reported that patients use reviews from different sources differently, and this makes it difficult to extrapolate how patients will view star ratings and comments posted by health systems [26]. At least one recent study suggests that patients trust commercial rating sites more than health systems’ sites [27].

One potential driver of this lack of trust is the finding that most (64%) health systems we examined stated on their main page that they remove “offensive or inappropriate content”; we also observed a relative lack of negative reviews compared to commercial physician-rating sites [12,25]. The finding that health system sites have more ratings, fewer negative ratings, and fewer negative comments compared to commercial physician-rating websites is consistent with the only published report that compared the two sources [13]. Despite the obvious possibility that negative comments are being removed by system administrators, there are several potential explanations for the observed differences. Removal of some comments is appropriate if the content is genuinely offensive [27], and patients may take a different approach to systematic surveys compared to open online platforms (which include reviews and comments only from respondents who seek out the site).

Our findings suggest that health systems' websites have the potential to provide patients with information about the experience of care with clinicians, but the sites may require improvements on this first iteration. One addition that could improve the narrative content is posting of a protocol for curating patient narratives and calculating star ratings [5-10]. Given the narrow distribution of the star ratings, a posting of the range of all physician scores with an indication where each physician’s score is situated would also be helpful (eg, if 4.2 is the lowest score, the patient would know this by seeing where the physician fell within the distribution). Although these improvements would provide patients with a more comprehensive picture of the experience of care with physicians within a given health system, it is also possible that health systems have conflicts of interest (specifically, an interest in increasing market share) that would discourage them from making these changes [11-12]. Another area that warrants further investigation—but about which we have limited information—is the possibility that the process of collecting and publishing the patient experience data has led physicians, practices, and even entire systems to initiate improvement activities based on patient comments [28,29].

This study has several limitations. First, we made extensive efforts to identify all health systems in the United States that are posting reviews of clinicians but may have missed some sites. Second, this is a snapshot of a single point in time, and the number of health systems participating in these efforts has likely changed in the interim. Third, we had a limited sample from which we drew clinician reviews for analysis because of the lack of lists of clinicians for most sites. However, we have no reason to believe that the health systems we sampled were different than the remaining health systems in our study. Finally, we were limited by an inability to assess the impact of these sites on clinicians or prospective patients.

Given the amount of public interest in narrative and quantitative data on individual clinicians, we anticipate that the trend of health systems publishing this information will continue. However, before health systems’ websites emerge as the main route by which consumers look for information about prospective clinicians, there may be a need to improve their methods used for curating and posting patient experience data.

## Appendix

Multimedia Appendix 1 Codebook.

Multimedia Appendix 2 Themes.

Multimedia Appendix 3 Example Quotations for Identified Themes.

## Abbreviations

CAHPS: Consumer Assessment of Healthcare Providers and Systems

## References

[1] Hanauer DA, Zheng K, Singer DC, Gebremariam A, Davis MM (2014). Public awareness, perception, and use of online physician rating sites. JAMA.

[2] Emmert M, Meier F, Pisch F, Sander U (2013). Physician choice making and characteristics associated with using physician-rating websites: cross-sectional study. J Med Internet Res.

[3] Lagu T, Metayer K, Moran M, Ortiz L, Priya A, Goff SL, Lindenauer PK (2017). Website characteristics and physician reviews on commercial physician-rating websites. JAMA.

[4] Lagu T, Hannon NS, Rothberg MB, Lindenauer PK (2010). Patients' evaluations of health care providers in the era of social networking: an analysis of physician-rating websites. J Gen Intern Med.

[5] Hays RD, Shaul JA, Williams VS, Lubalin JS, Harris-Kojetin LD, Sweeny SF, Cleary PD (1999). Psychometric properties of the CAHPS 1.0 survey measures. Consumer Assessment of Health Plans Study. Med Care.

[6] Giordano LA, Elliott MN, Goldstein E, Lehrman WG, Spencer PA (2010). Development, implementation, and public reporting of the HCAHPS survey. Med Care Res Rev.

[7] Grob R, Schlesinger M, Parker AM, Shaller D, Barre LR, Martino SC, Finucane ML, Rybowski L, Cerully JL (2016). Breaking narrative ground: innovative methods for rigorously eliciting and assessing patient narratives. Health Serv Res.

[8] Schlesinger M, Grob R, Shaller D, Martino SC, Parker AM, Finucane ML, Cerully JL, Rybowski L (2015). Taking patients' narratives about clinicians from anecdote to science. N Engl J Med.

[9] Schlesinger M, Kanouse DE, Martino SC, Shaller D, Rybowski L (2014). Complexity, public reporting, and choice of doctors: a look inside the blackest box of consumer behavior. Med Care Res Rev.

[10] Schlesinger M, Kanouse DE, Rybowski L, Martino SC, Shaller D (2012). Consumer response to patient experience measures in complex information environments. Med Care.

[11] Lee TH (2014). Harvard Business Review.

[12] Lee V (2017). Transparency and trust-online patient reviews of physicians. N Engl J Med.

[13] Ricciardi BF, Waddell BS, Nodzo SR, Lange J, Nocon AA, Amundsen S, Tarity TD, McLawhorn AS (2017). Provider-initiated patient satisfaction reporting yields improved physician ratings relative to online rating websites. Orthopedics.

[14] US Centers for Medicare & Medicaid Services (2010). Medicare.gov.

[15] National Research Council (2018). NRC Health.

[16] Lagu T, Kaufman EJ, Asch DA, Armstrong K (2008). Content of weblogs written by health professionals. J Gen Intern Med.

[17] Lagu T, Goff SL, Hannon NS, Shatz A, Lindenauer PK (2013). A mixed-methods analysis of patient reviews of hospital care in England: implications for public reporting of health care quality data in the United States. Jt Comm J Qual Patient Saf.

[18] Goff SL, Mazor KM, Gagne SJ, Corey KC, Blake DR (2011). Vaccine counseling: a content analysis of patient-physician discussions regarding human papilloma virus vaccine. Vaccine.

[19] Hsieh H, Shannon SE (2005). Three approaches to qualitative content analysis. Qual Health Res.

[20] (2017). Agency for Healthcare Research and Quality.

[21] López A, Detz A, Ratanawongsa N, Sarkar U (2012). What patients say about their doctors online: a qualitative content analysis. J Gen Intern Med.

[22] Greaves F, Laverty AA, Cano DR, Moilanen K, Pulman S, Darzi A, Millett C (2014). Tweets about hospital quality: a mixed methods study. BMJ Qual Saf.

[23] Ranard BL, Werner RM, Antanavicius T, Schwartz HA, Smith RJ, Meisel ZF, Asch DA, Ungar LH, Merchant RM (2016). Yelp reviews of hospital care can supplement and inform traditional surveys of the patient experience of care. Health Aff (Millwood).

[24] Kadry B, Chu LF, Kadry B, Gammas D, Macario A (2011). Analysis of 4999 online physician ratings indicates that most patients give physicians a favorable rating. J Med Internet Res.

[25] Algorithms for Innovation.

[26] Yaraghi N, Wang W, Gao GG, Agarwal R (2018). How online quality ratings influence patients' choice of medical providers: controlled experimental survey study. J Med Internet Res.

[27] Holliday AM, Kachalia A, Meyer GS, Sequist TD (2017). Physician and patient views on public physician rating websites: a cross-sectional study. J Gen Intern Med.

[28] Lagu T, Goff SL, Craft B, Calcasola S, Benjamin EM, Priya A, Lindenauer PK (2016). Can social media be used as a hospital quality improvement tool?. J Hosp Med.

[29] Emmert M, Meszmer N, Sander U (2016). Do health care providers use online patient ratings to improve the quality of care? results from an online-based cross-sectional study. J Med Internet Res.

