# Supplementary material for: Reporting of Patient Experience Data on Health Systems’ Websites and Commercial Physician-Rating Websites: Mixed-Methods Analysis
Source: J Med Internet Res. 2019 Mar 27;21(3):e12007. doi: 10.2196/12007 (PMC6456827; doi:10.2196/12007)
Supplement: Multimedia Appendix 1 [file jmir_v21i3e12007_app1.pdf]

Appendix 1: Codebook

1.0 POSITIVE COMMENTS

1.0 General positive comments about clinicians – *great doctor, very good, would recommend, satisfied with care from provider*

1.1. Clinician Communication

1.10 General positive about communication
1.11 Thorough
1.12 Listened well, attentive
1.13 Explained things clearly, informative, instructive
1.14 Included patient in decision-making regarding treatment plan
1.15 Followed up by phone or email; responded to phone calls/emails/patient portal messages
1.16 Spent enough time with patient or spent enough time taking care of patient's needs
1.17 Answered questions
1.18 Provided follow-up instructions
1.19 Remembered details of medical history

1.2. Clinician Interpersonal Skills

1.20 General positive about interpersonal skills
1.21 Positive personality traits – *likeable, kind, honest, trustworthy, nice, good sense of humor*
1.22 Good treatment of kids/family
1.23 Professionalism – *professional, non-judgmental, dedicated, dependable, respectful, patient, helpful, polite*
1.24 Reassuring – *put patient at ease, encouraging, patient has confidence in clinician*
1.25 Feels like a friend
1.26 Went above and beyond
1.27 Compassionate/caring – *demonstrates concern, good bedside manner*

1.3. Clinician Technical Skills

1.30 General positive about technical skills – *skilled, talented, competent*
1.31 Knowledgeable – *made correct diagnosis, ordered necessary tests*
1.32 Willing to refer if not certain of diagnosis
1.33 Efficient
1.34 Proactive
1.35 Has specific skill – *recommended to someone with the same issue, very good treatment of specific problem*
1.36 Took mental health into consideration, took mental health seriously

1.4. Facility and Office Experience

1.40 General positive about facility and office experience
1.41 Access – *easy to schedule appointments, good online portal*
1.42 Office characteristics – *good selection of magazines, well-decorated waiting room*
1.43 Wait time – *didn't have to wait long*
1.44 Cleanliness
1.45 Disability access
1.46 Fees were reasonable

1.5. Staff Characteristics

1.50 General positive about staff (nonspecific) - *"Staff was great"*
1.51 Medical staff –*nurses, PAs, medical assistants, residents*
1.52 Non-medical staff – *receptionists, billing office*

1.6. Patient Care Experience

1.60 General positive about patient care experience - *great experience, would recommend practice*

1.61 Positive comment about unspecified subjects- *Everyone was great, excellent team, includes doctors/fellows*

1.62 Better care experience compared to elsewhere

1.63 Privacy

1.64 Worth the drive

1.7. Reason for Seeking Care

1.70 General positive about recommendation

1.71 Recommended for interpersonal skills

1.72 Recommended for specialty/particular skill/experienced in a particular area

1.73 Sought care because of negative experience elsewhere\

1.8 Extremely Positive

1.81 Drastically improved life, saved life

1.82 Mention of God or religion – *God-given*
