# Supplementary material for: Reporting of Patient Experience Data on Health Systems’ Websites and Commercial Physician-Rating Websites: Mixed-Methods Analysis
Source: J Med Internet Res. 2019 Mar 27;21(3):e12007. doi: 10.2196/12007 (PMC6456827; doi:10.2196/12007)
Supplement: Multimedia Appendix 2 [file jmir_v21i3e12007_app2.pdf]

Reporting of Patient Experience Data on Health Systems' and Commercial Physician Rating Websites

Tara Lagu MD, MPH;[1,2] Caroline Norton, BS[3]; Lindsey Russo, MS[3]; Aruna Priya MA, MSc;[1,3] Sarah Goff, MD;[1,2,3] Peter K. Lindenauer MD, MSc[1,2]

1. Institute for Healthcare Delivery and Population Science, University of Massachusetts Medical School-Baystate, Springfield, MA
2. Department of Medicine, Baystate Medical Center, Springfield, MACollege of Natural Sciences, University of Massachusetts-Amherst, Amherst, MA
3. School of Public Health and Health Sciences, University of Massachusetts-Amherst, Amherst, MA

Article Type: Original Research Study

All authors had access to the data and wrote portions of the manuscript and reviewed the manuscript for content.

Funding Sources: Dr. Lagu is supported by the National Heart, Lung, and Blood Institute of the National Institutes of Health under Award Number K01HL114745.

Disclosures: The authors have no potential conflicts of interest. See COI for more details.

<u>Corresponding Author and Requests for Reprints:</u>
Tara Lagu MD, MPH
Institute for Healthcare Delivery and Population Science, Baystate Medical Center-University of Massachusetts Medical School, Springfield, MA
3601 Main St., 3rd Floor, Springfield, MA 01199
413-794-7688 (v); 215-756-2733 (mobile); lagutc@gmail.com

Word count for text only: 3,276

Tables: 3; Figures: 1; Appendices: 2

Key Words: Physician Reviews, Social Networking, Public Reporting

Running Head: Health Systems that Publish Physician Reviews

ABSTRACT

Background: Some hospitals' and health systems' websites report physician-level ratings and comments drawn from the Consumer Assessment of Healthcare Providers and Systems (CAHPS) surveys.

Objective: To examine the prevalence and content of health system websites reporting these data and compare narratives from these sites to narratives from commercial physician rating sites.

Methods: We identified health system websites active between June 1, 2016 and June 30, 2016 that posted clinician reviews. For 140 randomly-selected clinicians, we extracted the number of star ratings and narrative comments. We conducted a qualitative analysis of a random sample of these physicians' narrative reviews and compared to a random sample from commercial physician rating websites. We described composite quantitative scores for sampled physicians and compared frequency of themes between reviews drawn from health system and commercial physician rating websites.

Results: We identified 42 health systems that published composite star ratings (n=42, 100%) or narratives (n=33, 79%). Most (n=27, 64%) stated that they excluded narratives deemed offensive. Of 140 clinicians, a majority had composite scores listed: ("star ratings," n=122, 87%; narrative reviews, n = 114, 81%), with a median of 110 star ratings and 25 narratives. The median (IQR) rating was 4.8 (4.7 – 4.9) out of 5 stars and no clinician had a score below 4.2. Compared to commercial physician rating websites, we found significantly fewer negative comments on health system websites (36% vs. 13%, respectively, $P<0.001$).

Conclusion: The lack of variation in star ratings on health system sites may make it difficult to differentiate between clinicians. Most health systems report that they remove

“offensive” comments, and we notably found fewer negative comments on health system websites compared to commercial physician rating sites.

Introduction

Approximately 60% of U.S. consumers report that online reviews are either somewhat or very important when choosing a physician [1]. However, commercial

physician rating websites (designed similarly to websites that review restaurants and hotels) are difficult to use and have few reviews per physician [2,3]. Quantitative patient experience survey results are reported on some government and nonprofit public-reporting websites at the level of the hospital or the practice, but not at the level of the individual clinician. Research examining the feasibility of collecting and publishing systematically collected patient narratives about individual physicians is ongoing, but it is unclear when these initiatives will be broadly implemented [4-9]. Patients, therefore, may be seeking online sources of reviews and narratives about individual clinicians.

Both in response to this gap and as a mechanism to increase market share, some hospitals and health systems across the United States have begun to compile and report physician-level ratings and comments drawn from the Consumer Assessment of Healthcare Providers and Systems (CAHPS) surveys [10]. Typically, a health system engaging in such an effort summarizes CAHPS data through a composite score (hereafter called a "star rating") and posts this score on the physician's biographical webpage within the health system website. Many health systems also post patients' narrative responses to open-ended questions (e.g., "What did this clinician do well?" and "What could this clinician do better?"). We aimed to identify U.S. health systems that are participating in these efforts; to characterize the webpages that report these results, including numbers of star ratings and narrative reviews per clinician; and to compare the content of narrative comments drawn from commercial physician rating websites.

Methods

Search Strategy and Data Sources

Many health systems that publish compiled patient experience data are large systems associated with a hospital or hospitals. Therefore, we obtained a list of U.S.

hospitals from the Center for Medicare and Medicaid Services' *Hospital Compare* website and examined all listed hospitals. For each entity, we verified the name and street address and then examined the department pages as well as the biographical webpages of clinicians to determine if reviews were present. To identify participating health systems that were not associated with a hospital, we obtained a published online list of health systems [11] that have implemented this practice. We examined all sites on this list and also supplemented our search using a previously-described method for systematically searching Google (e.g., "Doctor reviews") [3].

We included websites that were functional (i.e., could be loaded and had working links) between June 1, 2016 and June 30, 2016 and had at least one clinician with star ratings or narrative comments. We used American Hospital Association data to generate hospital descriptive statistics (characteristics of health systems without a participating hospital were not captured). Because all included data were publicly available, the BMC Institutional Review Board deemed that this study did not constitute human subjects research.

Examination of Website Structure

Using an extraction method described previously, we created an *a priori* list of website classification criteria in order to describe included websites [3]. In brief, this included elements such as: methods that could be used to search for clinicians (e.g., specialty, name, location); description of methods used to remove "offensive" reviews; and review structure (e.g., star ratings vs. narratives). Three authors (TL, CN, LR) then reviewed content of the websites and added classification categories as appropriate. Two authors then completed a final review of included websites (CN, LR).

Creation of Physician Sample and Extraction of Physician-Review Data

In order to examine a sample of clinician reviews, we obtained, from the National Research Corporation (NRC) [12], lists of clinicians for 14 of the identified health systems. NRC is a for-profit consulting firm focused on improving patient experience and health system brand loyalty. We limited our sample to health systems for which we could obtain lists of clinicians because without a list we were unable to randomly select clinicians. Further, for websites that could not be easily searched, the absence of a list meant that we were unable to identify even a non-random sample of clinicians. We used random number generation to select 10 clinicians from each of the 14 identified health systems. We confirmed physician identity using the presence and confirmation of name (required fields: first name and last name plus either middle initial or subspecialty). For each clinician, we then extracted the number of star ratings, number of narrative comments, and total or average "star rating." We quantified occurrence of each type of review using descriptive statistics (frequencies and percentages).

Qualitative Analysis of Narrative Reviews.

We used qualitative methods to examine narrative reviews for included physicians. We selected the 5 most recent narratives from each of the 140 (10 clinicians from 14 health systems) randomly selected clinicians' profiles (if there were fewer than 5 narratives, we took as many as were present). Beginning with themes described in related studies [3,13,14], we created an *a priori* codebook. Initially, investigators met after coding every 20 comments in an effort to establish acceptable inter-rater agreement. Percent agreement was calculated as the number of matching codes within a block of text scored by both raters divided by the total number of codes. We developed additional codes to capture themes and content that was not in the *a priori* codebook. We repeated this iteratively until the team felt that the coding categories captured the major

substantive content reviewed. After establishing 80% agreement, the researchers each completed coding independently (All reviews were double-coded) and met once more in order to reach an agreement on all codes, resolving differences by consensus. Applying directed qualitative content analysis methods [15,16], we then organized codes into pertinent major and minor themes. One author (CN) then checked for accuracy of coding and performed second-level coding to synthesize themes and content, which was reviewed with the other authors. We used descriptive statistics to describe the frequency with which major and minor themes occurred.

Comparison of Narrative Reviews from Health System Websites to those from Commercial Physician Rating Websites

In a prior study of 600 physicians selected from 3 geographically diverse US cities, we collected over 1,800 narrative reviews from 28 different commercial physician rating websites.[2] We therefore conducted a simultaneous qualitative analysis of a randomly sampled set of 214 comments taken from these 28 commercial physician rating sites and compared the results to those obtained from our qualitative analysis of comments from health systems' websites. Using the codebook created for the analysis of reviews from health systems, the two coders coded commercial website reviews independently and then met to discuss discrepancies in coding. Investigators resolved differences in coding and updated the codebook using an iterative process. We continued this process until no new codes were identified in ten sequential reviews, resulting in a comprehensive codebook that covered both commercial rating websites and health systems (Appendix 1) and a comprehensive list of themes (Appendix 2). Two investigators (CN, LR) then independently coded the remaining reviews. We compared the percentage of reviews for each theme between health system and commercial rating

websites (both numbers over overall and by category) using the Chi-Square test and the Fisher's Exact test. All analyses were performed using the Statistical Analysis System (SAS Institute, Inc, Cary, NC. Version 9.4).

Results

Website Identification, Search Characteristics, and Hospital Characteristics

From 4800 hospitals on *Hospital Compare*, we identified 161 hospitals (3.4%) that posted star ratings or narrative comments about clinicians. Many of these hospitals were affiliated hospitals within a larger system, so we collapsed the 161 hospitals into 36 health systems. Our search methods identified an additional 8 health systems that were not associated with hospitals, which gave us a total of 42 health systems from 26 states (Figure 1). Of these, all health systems published star ratings (n=42, 100%) and most published narrative reviews (n=33, 79%). No sites described their method for calculating star ratings. The majority (n=27, 64%) stated on their main page that they excluded narratives deemed inappropriate or offensive, but none explained how this process was conducted. Most allowed users to search for physicians by name (n=39, 93%), specialty (n=41, 98%), and location (n=31, 74%). Nearly half of included hospitals (47%) had fewer than 200 beds (Table 1). More than a third (36%) were located in the western region of the US (Figure 1). Acute care hospitals made up the majority (86%) of the sample.

Quantity of Reviews

Of the randomly sampled 140 clinicians from 14 health systems, there were 21,332 quantitative reviews and 4,723 narrative reviews. A majority of clinicians had reviews (star ratings, n=122, 87%; narrative reviews, n = 114, 81%), with a median of 110 star ratings and 25 narratives per clinician. Only 1 clinician in the sample did not

have any reviews. In general, star ratings were quite high with little variation between physicians: the median rating was 4.8 (interquartile range [IQR] 4.7 – 4.9) out of 5 stars. Of 140 physicians, none had a score below 4.2.

Narrative Reviews

Using the five (or less if 5 were not available) most recent reviews from 140 clinicians from 14 health systems, we identified 561 health system narrative reviews for qualitative analysis. As described in the methods section, we also analyzed 214 narrative comments previously randomly sampled from 600 physicians across 28 commercial physician rating websites [2]. Themes that emerged from coding these two sets of data included general positive and negative comments about clinicians, clinician communication and interpersonal skills, technical skills, facility and office experience, patient care experience (independent of these other themes), descriptions of "reasons for seeking care," and "extreme comments" (i.e., long descriptions of very positive or negative experiences that did not fit well into other categories). Example quotes from these themes are given in Table 2.

Comparison of Occurrence by Theme for Commercial Physician Rating Websites and Health System Websites

Overall, the vast majority of comments were positive (82% for all narratives), including 71% of commercial rating websites comments and 87% of health systems websites comments (P <0.001). Negative comments were less common, but commercial rating sites (CS) had a greater proportion of negative reviews compared to health systems' (HS) sites (36% vs. 13%, respectively, P <0.001) (Table 3). Within subcategories of positive comments, there were some significant differences between the two types of websites. Commercial rating websites had significantly more "clinician

communication and personal skills” positive comments (CS n=127, 59%; HS n= 238, 42%; P <0.001), more positive “clinician technical skills” comments (CS n=74, 35%; HS n=84, 15%; P <0.001), and more “extremely positive” comments (CS n=9, 4%; HS n=4, 1%; P=0.002) compared to health system websites, while health system websites had significantly more positive “patient care experience” comments than commercial websites (CS n=12, 6%; HS n=106, 19%; P <0.001).

In contrast, commercial websites had a higher percentage of negative comments across nearly all themes. For example, commercial rating websites had more negative “clinician communication and interpersonal skills” comments (CS n=40, 19%; HS n= 32, 6%; P <0.001), “technical skills” comments (CS n=27, 13%; HS n=8, 1%; P <0.001), more “facility/office experience and staff characteristics” comments (CS n=38, 18%; HS n=28, 5%; P <0.001), “patient care experience” comments (CS n=16, 8%; HS n=8, 1%; P <0.001), and more “extremely negative” comments (CS n=13, 6%; HS n=0) compared to health system websites.

Discussion

After a comprehensive search for health systems that publish systematically collected patient experience surveys on the biographical webpages of individual clinicians, we identified a total of 42 health systems that are early adopters of this practice. Most clinicians’ pages had many reviews (both star reviews and narratives), which gives them an important advantage over existing commercial physician rating websites [2]. However, most clinicians also had near perfect star ratings, with 75% of physicians having a score of between 4.7 and 4.9 stars out of 5, and a minimum star rating of 4.2. Furthermore, we observed, similar to published literature [3,17], that narratives from both commercial-rating websites and health systems websites were

mostly positive (with similar percentages of positive responses across both types of sites), and themes that emerged were similar to themes seen in other studies that have examined review content [3,18, 19]. However, across a range of negative comment subthemes, we observed statistically significant differences in the number of reviews for individual clinicians on health system sites when compared to commercial physician rating sites.

We believe this is the first study to describe this phenomenon, and there are several important implications to our findings. First, the narrow range of star ratings (the majority were in the 4.7-4.9/5 range) may limit the ability of patients to differentiate between clinicians using only star ratings on health systems websites. The tightly clustered distribution of scores near 5.0 may be the result of the fact that health systems calculate the composite score from the CAHPS multi-question survey, and the majority of responses to CAHPS questions are either "usually" or "always.[4]" Less than 5% of respondents choose "never" for any CAHPS category.[4] However, it is notable that we did not find any health systems that explained their methods for calculating the composite metric of "number of stars."

Second, Hanauer et al recently reported that the majority of patients report that they are seeking reviews when looking for a physician [1]. Of note, Hanauer's questionnaire did not describe the type of reviews that patients were seeking. Because the question was posed to members of the general public, we interpret the word "reviews" to indicate the types of reviews available for restaurants and hotels: a star rating accompanied by a short narrative generated by the consumer. Thus, the large number of both star and narrative reviews we found on health system sites may meet this need. We have previously documented that reviews on any one commercial physician rating site are scarce: most sites have a median number of between 1 and 4 reviews per physician (after

the sample is limited to physicians with reviews) [2]. In contrast, we found that health systems posted a median of 110 star ratings and 25 narratives for each physician. This is likely because CAHPS surveys are sent to a large number of patients, so there are more responses per clinician. Health systems can also wait to post reviews until a sufficient number are collected or can use older surveys to increase the numbers of reviews and narratives. For example, the University of Utah reports that they only post reviews for physicians with more than 30 responses in a year [20,21].

There are some caveats that go along with the increased number of reviews on health system sites. The relative lack of negative reviews compared to commercial physician rating sites suggests that health systems may have opted not to post a relatively large proportion of negative reviews. This is consistent with the finding that 64% of health systems we examined stated on their main page that they remove “offensive or inappropriate content” and consistent with reports from the health systems themselves [20,21]. Removal of some comments is appropriate if the content is genuinely offensive. Indiscriminate posting of offensive or inappropriate material is one of the main complaints that physicians have stated about commercial physician rating sites [22]. The way that systems defined “offensive” is not clearly stated by the health systems, however. Some patients who find these sites may not be aware that not all comments are included, but others may be more savvy: a recent study suggests that patients trust commercial rating sites more than health systems’ sites [22].

We must also consider the possibility that the removal of negative comments from health systems’ sites was not the driver of the differences observed in the content on the two types of sites. Rather, the differences could be the result of patients’ differing approaches to systematic surveys vs. open online platforms (which take a haphazard

approach and include reviews and comments only from respondents who seek out the site).

Our findings suggest that health system websites have the potential to provide patients with the information about the experience of care with clinicians, but the sites may require improvements on this first iteration. One addition that could improve the narrative content is posting of a published protocol for curating patient narratives and calculating star ratings [4-9]. Given the narrow distribution of the star ratings, a new method for calculating the composite scores that allows for more variation across clinicians would also provide patients with more information. If this were not possible, a posting of the range of all physician scores with an indication where each physician's score is situated would be helpful (e.g., if 4.2 is the lowest score, the patient would know this by seeing where the physician fell within the distribution). While these improvements would provide patients with a fuller picture of the experience of care with physicians within a given health system, it is also possible that health systems have conflicts of interest (specifically, an interest in increasing market share) that would discourage them from making these changes [10-21].

This study has several limitations. First, we made extensive efforts to identify all health systems in the United States that are posting reviews of clinicians, but may have missed some sites. Second, this is a snapshot of a single point in time, and the number of health systems participating in these efforts has likely changed in the interim. Third, we had a limited sample from which we drew clinician reviews for analysis because of the lack of lists of clinicians for most sites. We have no reason to believe, however, that the health systems we sampled were different than the remaining health systems in our study.

Finally, we were limited by an inability to assess the impact of these sites on prospective patients or clinicians.

Given the amount of public interest in narrative and quantitative data on individual clinicians, we anticipate that the trend of health systems publishing this information will continue. However, the limitations of these sites may prevent them from emerging as the route by which consumers are most likely to look for information about the experience of care with a prospective clinician.

Table and Figure Legend

Table 1: Characteristics of 169 hospitals posting physician reviews

Table 2: Example Quotations for Identified Themes

Table 3: Comparison of Occurrence of Themes Between Health Systems Sites and Commercial Ratings Sites

Figure 1: Geographic location of included health systems located in the 48 contiguous United States

Appendix 1: Codebook

Appendix 2: Themes

Bibliography

1. Hanauer DA, Zheng K, Singer DC, Gebremariam A, Davis MM. PUblic awareness, perception, and use of online physician rating sites. *JAMA*. 2014;311(7):734-735. doi:10.1001/jama.2013.283194

2. Lagu T, Metayer K, Moran M, et al. Website Characteristics and Physician Reviews on Commercial Physician-Rating Websites. *JAMA*. 2017;317(7):766-768. doi:10.1001/jama.2016.18553
3. Lagu T, Hannon NS, Rothberg MB, Lindenauer PK. Patients' evaluations of health care providers in the era of social networking: an analysis of physician-rating websites. *J Gen Intern Med*. 2010;25(9):942-946. doi:10.1007/s11606-010-1383-0
4. Hays RD, Shaul JA, Williams VS, et al. Psychometric properties of the CAHPS 1.0 survey measures. Consumer Assessment of Health Plans Study. *Med Care*. 1999;37(3 Suppl):MS22-31.
5. Giordano LA, Elliott MN, Goldstein E, Lehrman WG, Spencer PA. Development, Implementation, and Public Reporting of the HCAHPS Survey. *Medical Care Research and Review*. 2010;67(1):27-37. doi:10.1177/1077558709341065.
6. Grob R, Schlesinger M, Parker AM, et al. Breaking Narrative Ground: Innovative Methods for Rigorously Eliciting and Assessing Patient Narratives. *Health Serv Res*. April 2016. doi:10.1111/1475-6773.12503
7. Schlesinger M, Grob R, Shaller D, et al. Taking Patients' Narratives about Clinicians from Anecdote to Science. *N Engl J Med*. 2015;373(7):675-679. doi:10.1056/NEJMsb1502361
8. Schlesinger M, Kanouse DE, Martino SC, Shaller D, Rybowski L. Complexity, public reporting, and choice of doctors: a look inside the blackest box of consumer behavior. *Med Care Res Rev*. 2014;71(5 Suppl):38S-64S. doi:10.1177/1077558713496321
9. Schlesinger M, Kanouse DE, Rybowski L, Martino SC, Shaller D. Consumer response to patient experience measures in complex information environments. *Med Care*. 2012;50 Suppl:S56-64. doi:10.1097/MLR.0b013e31826c84e1
10. Lee TH, MD. Online Reviews Could Help Fix Medicine. Harvard Business Review. https://hbr.org/2014/06/online-reviews-could-help-fix-medicine. Accessed November 25, 2014.
11. Centers for Medicare and Medicaid Services. Hospital Compare. http://www.hospitalcompare.hhs.gov. Published 2010. Accessed April 19, 2010.
12. National Research Council. NRC Health. https://nrchealth.com/. Accessed February 12, 2018.
13. Lagu T, Kaufman EJ, Asch DA, Armstrong K. Content of weblogs written by health professionals. *J Gen Intern Med*. 2008;23(10):1642-1646. doi:10.1007/s11606-008-0726-6
14. Lagu T, Goff SL, Hannon NS, Shatz A, Lindenauer PK. A mixed-methods analysis of patient reviews of hospital care in England: implications for public reporting of health care quality data in the United States. *Jt Comm J Qual Patient Saf*. 2013;39(1):7-15.
15. Goff SL, Mazor KM, Gagne SJ, Corey KC, Blake DR. Vaccine counseling: a content analysis of patient-physician discussions regarding human papilloma virus vaccine. *Vaccine*. 2011;29(43):7343-7349. doi:10.1016/j.vaccine.2011.07.082
16. Hsieh H-F, Shannon SE. Three Approaches to Qualitative Content Analysis. *Qualitative Health Research*. 2005;15(9):1277-1288. doi:10.1177/1049732305276687

17. López A, Detz A, Ratanawongsa N, Sarkar U. What patients say about their doctors online: a qualitative content analysis. *J Gen Intern Med*. 2012;27(6):685-692. doi:10.1007/s11606-011-1958-4
18. Greaves F, Laverty AA, Cano DR, et al. Tweets about hospital quality: a mixed methods study. *BMJ Qual Saf*. 2014;23(10):838-846. doi:10.1136/bmjqs-2014-002875
19. Ranard BL, Werner RM, Antanavicius T, et al. Yelp Reviews Of Hospital Care Can Supplement And Inform Traditional Surveys Of The Patient Experience Of Care. *Health Aff (Millwood)*. 2016;35(4):697-705. doi:10.1377/hlthaff.2015.1030
20. TRANSPARENCY: WILL IT HELP OR HARM HEALTH CARE? http://uofuhealth.utah.edu/innovation/algorithms/2015/three.php. Accessed May 21, 2018.
21. Lee V. Transparency and Trust - Online Patient Reviews of Physicians. *N Engl J Med*. 2017;376(3):197-199. doi:10.1056/NEJMp1610136
22. Holliday AM, Kachalia A, Meyer GS, Sequist TD. Physician and Patient Views on Public Physician Rating Websites: A Cross-Sectional Study. *J Gen Intern Med*. 2017;32(6):626-631. doi:10.1007/s11606-017-3982-5

Table 1: Characteristics of 169 hospitals posting physician reviews

		Number of Hospitals n (%)
	Total	169 (100.0)
Number of Beds*		
<200		79 (46.8)
200 to 400		43 (25.4)
>400		25 (14.8)
Teaching*		20 (11.8)
Region*		
Midwest		25 (14.8)
Northeast		27 (16.0)
South		34 (20.1)
West		61 (36.1)
Hospital type **		
Acute care		145 (85.8)
Childrens'		2 (1.2)
Critical Access		15 (8.9)

*22 of the 169 hospitals do not have an AHA identifier
**7 of the 169 hospitals do not have their type/ownership recorded

Table 2: Example Quotations for Identified Themes

Theme	Source	Positive Quotation	Negative Quotation
**General Comments about Clinicians**	Health Systems	“My favorite care provider!”	“Perfume and body cologne can affect patients’ breathing.”
		“Love all my doctors. They are the best I have ever been too.”	
		“I would definitely recommend Dr. X to my friends [she is very thorough].”	
	Commercial Ratings	“Excellent Doctor totally love him”	“If you want to stay healthy and make good decisions about your health, avoid Dr. X at all costs.”
		“I highly recommend this doctor.”	“I couldn't recommend her and feel good about it.”
		“Dr. X is wonderful. She is young and has only been in practice a few years however, don't let that fool you.”	“He was totally useless to me. Whoever is paying him is a fool.”
**Clinician Communication and Interpersonal Skills**	Health System	“Very professional and courteous.”	“The doctor, actually read and addressed every one of the reasons for scheduling an appointment.”
		“She [is highly professional	“The doctor needs to spend

		and] is a great listener; she quickly understands the heart of the issue."	more time with me than the on average 8 minutes we patients get."
		"She is warm and personable and deeply compassionate"	"He walked in the door and quickly told me he was not going to see me. I've been tossed from doctor to doctor with my medical conditions being ignored."
	Commercial Ratings	"Dr. X is the most gentle, caring doctor."	"[I like Doctor X], but he tends to hurry the exams to get on to the next patient"
		"She treats her patients with respect. Without her, I'm not sure I'd be here."	"Steer clear of this one. Shop around. His demeanor is not professional, clearly he smirks and laughs at any question you have."
		"He eased my fears and frustrations. I don't think I have had any Dr. do that!"	"The snide remarks made by both [nurses and doctor] is unprofessional and made me feel very Uncomfortable."
**Clinician Technical Skills**	Health System	"He seems to be on top of having all the information I need."	"Problem hooking up EKG & she had to change it. She was very rough taking them off."
		"Dr. X seems to be well versed in the knowledge of his specialty"	"She didn't know why I was here and kept saying you will be fine"
		"Dr. X is the most competent, [personable] doctor I have."	"Not my regular provider. She kept insisting my chest pain had nothing to do with my heart. I have Hypertrophic Cardiomyopathy and pain is usual and common."
	Commercial Ratings	"And, he's probably the first doctor really treating me properly for my diagnosis and symptoms"	"Knowledge outside heart is poor (my child asked him what another major organ did and he could not answer then chuckled about his lack of knowledge)."
		"Dr. X is VERY knowledgeable and knows his stuff!"	"According to 2 other doctors - he ran excessive tests that were not needed. I have to wonder, were they so his office could pocket big bucks?"

		Had an incredibly difficult surgery but he did everything that needed to be done and I'm still here! He also took a skin graft that was so well done I can't even see where it was now"	"All they do is refer you out to other doctors, [you never can get an appointment] always had to go to hospital, I changed doctors…should have changed sooner."
**Facility/Office Experience and Staff Characteristics**	Health Systems	"The staff is always friendly and remember your name."	"Wait times for labs are unacceptable. The past 2 times, I've waited 1 hous for labs to be drawn."
		"Excellent facilities and staff."	"I was mis-scheduled by the office & then waited several hours to be seen."
		"All his assistants have always been kind & understanding.	His staff leaves something to be desired."
	Commercial Ratings	"The entire staff is very good. They return calls, answer emails, complete refills and go the extra mile to accommodate their patients."	"No one returns calls"
		"Her assistants were kind and funny keeping the experience light and, dare I say, somewhat enjoyable"	"The room I was taken into was not cleaned after the previous patient."
		"Her staff is filled with caring people who take every effort to make you feel comfortable."	"With such a rude staff, it's not worth the hassle anymore."
**Patient Care Experience**	Health Systems	"Everyone was courteous and helpful. Very patient and kind."	"The other doctors deferred to the first resident's decision, and weren't interested in communicating with me. It was a pretty serious infection, and the residents' plan, which they stuck with, was inadequate to treat it."
		"This was a very good experience."	"[Great facility], but insurance frustrations."
		"Besides the surgery clinic. I also use the Eye Clinic, the Dermatology Clock and the Urology Clinic, and am satisfied with the care from each of these."	"Don't go there."

	Commercial Ratings	“My experience was exceptional.”	“They ALWAYS collected my co-pay though I only came in for routine/preventive physicals that are covered at 100% by my insurance. Okay, whatever.”
		“My prior physician never informed me of my results...had to contact him myself 2 or 3 times in order to get a response…”	“I've worked in the medical field for 4 years and feel it's my duty to forewarn potential patients not to go to this facility!!! This place was totally unprofessional from the start...Needless to say, I left without being seen. It was overall the worst clinic experience I could imagine and so unethical.”
		“I couldn't be more satisfied with our experience with [her, and] Lexington Pediatrics as a whole.”	“Unbelievable this practice is open.”
**Reason for Seeking Care**	Health Systems	“Have heard many good things about him from past patients.”	
		“He came recommended to us from a friend whose child had the same issues as ours.”	
	Commercial Ratings	“This pediatrician was recommended to me by OBGYN when I was pregnant.”	
		“I had a fairly rare condition occur and my Father recommended I see Dr. X, his Urologist.	
**Extreme (Very Positive or Negative) Comments**	Health Systems	“Dr. X is the best OBGYN I have seen. She was fantastic during my entire pregnancy on monitoring me!..My c-section was a breeze! She is fantastic!!”	.”
		“In short, she is my dream doctor. She is brilliant, [compassionate, ethical and determined. She treats her patients with respect]. Without her, I'm not sure I'd be here. She is a star! I'm so grateful …”	

	Commercial Ratings	"Dr. X saved my little girl. Made us feel like we were his only patients. I would recommend him to anyone who wants the highest quality care with a genuinely helpful, knowledgeable, and compassionate high-risk OB."	"We exchanged words and I left his office only to have him chase me down and try to provoke a physical confrontation with me, on camera no less."
		Dr X is a truly amazing doctor and surgeon. I would recommend him to anyone in need of a great doctor. He has an awesome bedside manner and spends time with you as if you are his only patient. He is very prompt and spends as much or as little time with you as you need. He truly saved my life!!	"One of the most unprofessional and detached doctors I have ever come across. Because of her incompetence and wrong 'advice', we had to suffer health concerns which are still under treatment till date. We have already reported to the hospital and will also be reporting her to the state board."

Table 3: Comparison of Occurrence of Themes Between Health Systems Sites and Commercial Ratings Sites

Themes	Total	Commercial Rating Sites	Health Systems Sites	
	N (%)	N (%)	N (%)	*P*-value*
	775 (100)	214 (27.6)	561 (72.4)	
**Positive Themes**				
Overall positive comments	642 (82.8)	152 (71.0)	490 (87.3)	<0.001
General positive comments about clinicians – *great doctor, very good, would recommend, satisfied with care from provider*	345 (44.5)	89 (41.6)	256 (45.6)	0.31
Clinician Communication and Interpersonal Skills	365 (47.1)	127 (59.3)	238 (42.4)	<0.001
Clinician Technical Skills	158 (20.4)	74 (34.6)	84 (15.0)	<0.001
Facility/Office Experience and Staff Characteristics	127 (16.4)	31 (14.5)	96 (17.1)	0.38
Patient Care Experience	118 (15.2)	12 (5.6)	106 (18.9)	<0.001
Reason for Seeking Care	5 (0.6)	2 (0.9)	3 (0.5)	0.62^
Extremely Positive	13 (1.7)	9 (4.2)	4 (0.7)	0.002^
**Negative Themes**				
Overall negative comments	148 (19.1)	76 (35.5)	72 (12.8)	<0.001
General negative comments about clinicians- *would not recommend*	12 (1.5)	11 (5.1)	1 (0.2)	<0.001^
Clinician Communication and Interpersonal Skills	72 (9.3)	40 (18.7)	32 (5.7)	<0.001
Technical Skills	35 (4.5)	27 (12.6)	8 (1.4)	<0.001
Facility/Office Experience and Staff Characteristics	66 (8.5)	38 (17.8)	28 (5.0)	<0.001
Patient Care Experience	24 (3.1)	16 (7.5)	8 (1.4)	<0.001

Feedback about Survey	7 (0.9)	0 (0)	7 (1.2)	-
Extremely Negative	13 (1.7)	13 (6.1)	0 (0)	-
**Neutral Themes**				
Neutral patient experience	86 (11.1)	22 (10.3)	64 (11.4)	0.65

*Chi-Square test
^Fisher's exact test

Figure 1: Geographic location of included health systems located in the 48 contiguous United States

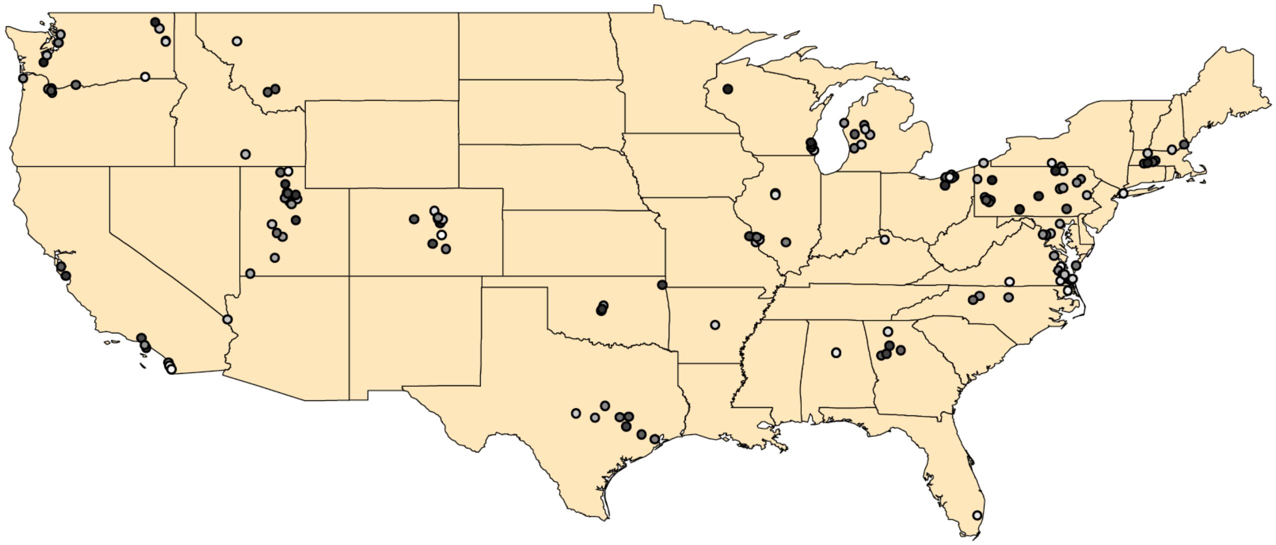

Appendix 1: Codebook

1.0 POSITIVE COMMENTS

1.0 General positive comments about clinicians – *great doctor, very good, would recommend, satisfied with care from provider*

1.1. Clinician Communication

1.10 General positive about communication
1.11 Thorough
1.12 Listened well, attentive
1.13 Explained things clearly, informative, instructive
1.14 Included patient in decision-making regarding treatment plan
1.15 Followed up by phone or email; responded to phone calls/emails/patient portal messages
1.16 Spent enough time with patient or spent enough time taking care of patient's needs
1.17 Answered questions
1.18 Provided follow-up instructions
1.19 Remembered details of medical history

1.2. Clinician Interpersonal Skills

1.20 General positive about interpersonal skills
1.21 Positive personality traits – *likeable, kind, honest, trustworthy, nice, good sense of humor*
1.22 Good treatment of kids/family
1.23 Professionalism – *professional, non-judgmental, dedicated, dependable, respectful, patient, helpful, polite*
1.24 Reassuring – *put patient at ease, encouraging, patient has confidence in clinician*
1.25 Feels like a friend
1.26 Went above and beyond
1.27 Compassionate/caring – *demonstrates concern, good bedside manner*

1.3. Clinician Technical Skills

1.30 General positive about technical skills – *skilled, talented, competent*
1.31 Knowledgeable – *made correct diagnosis, ordered necessary tests*
1.32 Willing to refer if not certain of diagnosis
1.33 Efficient
1.34 Proactive

1.35 Has specific skill – *recommended to someone with the same issue, very good treatment of specific problem*
1.36 Took mental health into consideration, took mental health seriously

1.4. Facility and Office Experience
1.40 General positive about facility and office experience
1.41 Access – *easy to schedule appointments, good online portal*
1.42 Office characteristics – *good selection of magazines, well-decorated waiting room*
1.43 Wait time – *didn't have to wait long*
1.44 Cleanliness
1.45 Disability access
1.46 Fees were reasonable

1.5. Staff Characteristics
1.50 General positive about staff (nonspecific) - *"Staff was great"*
1.51 Medical staff –*nurses, PAs, medical assistants, residents*
1.52 Non-medical staff – *receptionists, billing office*

1.6. Patient Care Experience
1.60 General positive about patient care experience - *great experience, would recommend practice*
1.61 Positive comment about unspecified subjects- *Everyone was great, excellent team, includes doctors/fellows*
1.62 Better care experience compared to elsewhere
1.63 Privacy
1.64 Worth the drive

1.7. Reason for Seeking Care
1.70 General positive about recommendation
1.71 Recommended for interpersonal skills
1.72 Recommended for specialty/particular skill/experienced in a particular area
1.73 Sought care because of negative experience elsewhere\

1.8 Extremely Positive
1.81 Drastically improved life, saved life
1.82 Mention of God or religion – *God-given*

Appendix 2: Themes

POSITIVE COMMENTS

A. General positive comments about clinicians

B. Clinician Communication and Interpersonal Skills

C. Clinician Technical Skills

D. Facility/Office Experience and Staff Characteristics

E. Patient Care Experience

X. Reason for Seeking Care

Y. Extremely Positive

NEGATIVE COMMENTS

F. General negative comments about clinicians- *would not recommend*

G. Clinician Communication and Interpersonal Skills

H. Technical Skills

I. Facility/Office Experience and Staff Characteristics

J. Patient Care Experience

Z. Extremely Negative
