# Supplementary material for: Reporting of Patient Experience Data on Health Systems’ Websites and Commercial Physician-Rating Websites: Mixed-Methods Analysis
Source: J Med Internet Res. 2019 Mar 27;21(3):e12007. doi: 10.2196/12007 (PMC6456827; doi:10.2196/12007)
Supplement: Multimedia Appendix 3 [file jmir_v21i3e12007_app3.pdf]

Multimedia Appendix 3: Example Quotations for Identified Themes

Theme	Source	Positive Quotation	Negative Quotation
**General Comments about Clinicians**	Health Systems	"My favorite care provider!"	"Perfume and body cologne can affect patients' breathing."
		"Love all my doctors. They are the best I have ever been too."	
		"I would definitely recommend Dr. X to my friends<br><br>[she is very thorough]."	
	Commercial Ratings	"Excellent Doctor totally love him"	"If you want to stay healthy and make good decisions about your health, avoid Dr. X at all costs."
		"I highly recommend this doctor."	"I couldn't recommend her and feel good about it."
		"Dr. X is wonderful. She is young and has only been in practice a few years however, don't let that fool you."	"He was totally useless to me. Whoever is paying him is a fool."
**Clinician Communication and Interpersonal Skills**	Health System	"Very professional and courteous."	"The doctor, actually read and addressed every one of the reasons for scheduling an appointment."
		"She [is highly professional and] is a great listener; she quickly understands the heart of the issue."	"The doctor needs to spend more time with me than the on average 8 minutes we patients get."
		"She is warm and personable and deeply compassionate"	"He walked in the door and quickly told me he was not going to see me. I've been tossed from doctor to doctor with my medical conditions being ignored."

	Commercial Ratings	“Dr. X is the most gentle, caring doctor.”	“[I like Doctor X], but he tends to hurry the exams to get on to the next patient”
		“She treats her patients with respect. Without her, I'm not sure I'd be here.”	“Steer clear of this one. Shop around. His demeanor is not professional, clearly he smirks and laughs at any question you have.”
		“He eased my fears and frustrations. I don't think I have had any Dr. do that!”	“The snide remarks made by both [nurses and doctor] is unprofessional and made<br><br>me feel very Uncomfortable.”
**Clinician Technical Skills**	Health System	“He seems to be on top of having all the information I need.”	“Problem hooking up EKG & she had to change it. She was very rough taking them off.”
		“Dr. X seems to be well versed in the knowledge of his specialty”	“She didn't know why I was here and kept saying you will be fine”
		“Dr. X is the most competent, [personable] doctor I have.”	“Not my regular provider. She kept insisting my chest pain had nothing to do with my heart. I have Hypertrophic Cardiomyopathy and pain is usual and common.”
	Commercial Ratings	“And, he's probably the first doctor really treating me properly for my diagnosis and symptoms”	“Knowledge outside heart is poor (my child asked him what another major organ did and he could not answer then chuckled about his lack of knowledge).”
		“Dr. X is VERY knowledgeable and knows his stuff!”	“According to 2 other doctors - he ran excessive tests that were not needed. I have to wonder, were they so his office could pocket big bucks?”
		Had an incredibly difficult surgery but he did everything that needed to be done and I'm still here! He also took a skin graft that was so well done I can't even see where it was now”	“All they do is refer you out to other doctors, [you never can get an appointment] always had to go to hospital, I changed doctors…should have changed sooner.”
**Facility/Office**	Health	“The staff is always friendly and remember your name.”	“Wait times for labs are

**Experience and Staff Characteristics**	Systems		unacceptable. The past 2 times, I've waited 1 hous for labs to be drawn.”
		“Excellent facilities and staff.”	“I was mis-scheduled by the office & then waited several hours to be seen.”
		“All his assistants have always been kind & understanding.	His staff leaves something to be desired.”
	Commercial Ratings	“The entire staff is very good. They return calls, answer emails, complete refills and go the extra mile to accommodate their patients.”	“No one returns calls”
		“Her assistants were kind and funny keeping the experience light and, dare I say, somewhat enjoyable”	“The room I was taken into was not cleaned after the previous patient.”
		“Her staff is filled with caring people who take every effort to make you feel comfortable.”	“With such a rude staff, it's not worth the hassle anymore.”
**Patient Care Experience**	Health Systems	“Everyone was courteous and helpful. Very patient and kind.”	“The other doctors deferred to the first resident's decision, and weren't interested in communicating with me. It was a pretty serious infection, and the residents' plan, which they stuck with, was inadequate to treat it.”
		“This was a very good experience.”	“[Great facility], but insurance frustrations.”
		“Besides the surgery clinic. I also use the Eye Clinic, the Dermatology Clock and the Urology Clinic, and am satisfied with the care from each of these.”	“Don't go there.”
	Commercial Ratings	“My experience was exceptional.”	“They ALWAYS collected my co-pay though I only came in for routine/preventive physicals that are covered at 100% by my insurance. Okay, whatever.”

		“My prior physician never informed me of my results...had to contact him myself 2 or 3 times in order to get a response…”	“I've worked in the medical field for 4 years and feel it's my duty to forewarn potential patients not to go to this facility!!! This place was totally unprofessional from the start...Needless to say, I left without being seen. It was overall the worst clinic experience I could imagine and so unethical.”
		“I couldn't be more satisfied with our experience with [her, and] Lexington<br><br>Pediatrics as a whole.”	“Unbelievable this practice is open.”
**Reason for Seeking Care**	Health Systems	“Have heard many good things about him from past patients.”	
		“He came recommended to us from a friend whose child had the same issues as ours.”	
	Commercial Ratings	“This pediatrician was recommended to me by OBGYN when I was pregnant.”	
		“I had a fairly rare condition occur and my Father recommended I see Dr. X, his Urologist.	
**Extreme (Very Positive or Negative) Comments**	Health Systems	“Dr. X is the best OBGYN I have seen. She was fantastic during my entire pregnancy on monitoring me!..My c-section was a breeze! She is fantastic!!”	.”
		“In short, she is my dream doctor. She is brilliant, [compassionate, ethical and determined. She treats her patients with respect]. Without her, I'm not sure I'd be here. She is a star! I'm so grateful …”	
	Commercial Ratings	“Dr. X saved my little girl. Made us feel like we were his only patients. I would recommend him to anyone who wants the highest quality care with a genuinely helpful,	“We exchanged words and I left his office only to have him chase me down and try to provoke a physical confrontation with me, on camera no less.”

		knowledgeable, and compassionate high-risk OB.”	
		Dr X is a truly amazing doctor and surgeon. I would recommend him to anyone in need of a great doctor. He has an awesome bedside manner and spends time with you as if you are his only patient. He is very prompt and spends as much or as little time with you as you need. He truly saved my life!!	“One of the most unprofessional and detached doctors I have ever come across. Because of her incompetence and wrong 'advice', we had to suffer health concerns which are still under treatment till date. We have already reported to the hospital and will also be reporting her to the state board.”
